# Supplementary material for: Content-rich biological network constructed by mining PubMed abstracts
Source: BMC Bioinformatics. 2004 Oct 8;5:147. doi: 10.1186/1471-2105-5-147 (PMC528731; doi:10.1186/1471-2105-5-147)
Supplement: Additional File 2 — The original results of the above study (non-essential files are deleted to keep the file size under the limit set by BMC bioinformatics). [file 1471-2105-5-147-S2.bz2 › chilibotAdditionalFile2/dip20/420ID7890768E10289/html/ACVR2_INHA.html]

 


 **ACVR2** and **INHA** 
  
Found 28 abstracts in PubMed, retrieved 20.  
 

 What does Google say? 
 PDF only 
| .edu only 

---

**Interactive relationship** (e.g. stimulation, inhibition, etc)

**Non-interactive relationship** (e.g. studied together, co-existance, homology, etc.)

- METHODS BPH tissues were obtained at surgery, and the mRNA expression for the inhibin alpha  [ **INHA** ] , beta A, beta B subunits, the putative activin beta C subunit, the activin type II receptor ActRII  [ **ACVR2** ] , and the activin binding protein, follistatin, was determined by reverse transcription polymerase chain reaction RT PCR and Southern blot analysis.  Ref: 9428386 Prostate, 1998
- Since peritoneal fluid hormone content may reflect in part ovarian and endometrial secretory activities, the present study aimed to evaluate i whether inhibin alpha  [ **INHA** ] , activin betaA and betaB subunits, and activin receptor type II  [ **ACVR2** ]  and type IIB mRNA are expressed in peritoneal tissues.  Ref: 9806293 Hum Reprod, 1998
- Complementary DNA samples produced by reverse transcription were used in the real time PCR to quantify the expression of inhibin alpha  [ **INHA** ]  subunit, inhibin activin beta A, beta B, beta C subunits, follistatin, ACTRIA, ACTRIB, ACTRIIA  [ **ACVR2** ] , ACTRIIB, beta glycan and GAPDH genes.  Ref: 12651901 Mol Hum Reprod, 2003
- These different responses to parietal cell loss suggest that stimulation of ActRII  [ **ACVR2** ]  dependent signaling pathways in  **inha**  m1 m1 animals affects the proliferative activity of the stem cell and its immediate descendents.  Ref: 9482661 Mol Endocrinol, 1998
- **inha**  m1 m1 actRII  [ **ACVR2** ]  m1 m1 compound homozygotes develop activin secreting gonadal tumors but have no abnormalities in their gastric epithelium, indicating that persistent stimulation of ActRII  [ **ACVR2** ]  dependent signaling pathways produces pleiotrophic effects on gastric epithelial differentiation.  Ref: 9482661 Mol Endocrinol, 1998
- By RT PCR, the expression of specific messenger RNAs mRNAs for the inhibin alpha  [ **INHA** ] , activin betaA and betaB subunits, and activin receptor type II  [ **ACVR2** ]  and type IIB was investigated 1 in primary cultures of endometrial stroma and epithelium or cervical epithelium cells from healthy women.  Ref: 9543140 J Clin Endocrinol Metab, 1998
- Our findings that messenger RNAs encoding inhibin alpha  [ **INHA** ]  subunit, inhibin activin beta A subunit, and activin receptor  [ **ACVR2** ]  II were expressed, and inhibin activin proteins were produced by MCF 7 cells, imply that these gonadal growth factors may have paracrine autocrine functions in human breast cancer.  Ref: 8750582 Breast Cancer Res Treat, 1996
- The objective of this study was to determine effects of postnatal age on expression of follistatin, inhibin alpha  [ **INHA** ]  subunit, betaA subunit, betaB subunit, and activin receptors Type IA ActRIA, Type IB ActRIB and Type II ActRII  [ **ACVR2** ]  in the developing ovine uterus.  Ref: 12748120 Biol Reprod< MedlineTA>Biol Reprod, 1996
- Cultured endometrial stromal or epithelial cells and epithelial cervical cells expressed inhibin alpha  [ **INHA** ] , activin betaA and betaB, and activin receptor type II  [ **ACVR2** ]  and type IIB mRNAs.  Ref: 9543140 J Clin Endocrinol Metab, 1998
- All six cell lines contained abundant messenger RNA mRNA for activin receptor type II  [ **ACVR2** ] , but no inhibin alpha  [ **INHA** ]  subunit mRNA was detected in any cell line.  Ref: 8593793 Endocrinology, 1996
